# Impact of BaO on the gamma-ray shielding performance of lanthanum barium-borate glasses

**DOI:** 10.1038/s41598-025-13248-0

**Published:** 2025-10-23

**Authors:** M. I. Sayyed, Chaitali V. More, Mohamed Y. Hanfi, Sudha D. Kamath

**Affiliations:** 1 Department of Physics, Faculty of Science, Isra University, Amman, Jordan; 2 Renewable Energy and Environmental Technology Center, University of Tabuk, Tabuk, 47913 Saudi Arabia; 3 Department of Physics and Technical Sciences, Western Caspian University, Baku, Azerbaijan; 4 Department of Physics, Dr. Babasaheb Ambedkar Marathwada University, Chhatrapati Sambhajinagar (MS), 431004 India; 5 Department of Physics, Government Institute of Forensic Science, Chhatrapati Sambhajinagar (MS), 431004 India; 6 Department of Life Safety, Institute of Fundamental Education, Ural Federal University, Ekaterinburg, 620002 Russia; 7 Nuclear Materials Authority, P.O. Box 530, El-Maadi, Cairo, Egypt; 8 Department of Physics, Manipal Institute of Technology, Manipal Academy of Higher Education, Manipal, Karnataka India

**Keywords:** Gamma radiation shielding, Barium-lanthanum borate glasses, Lead-free materials, Mass attenuation coefficient, Effective atomic number, Materials science, Physics

## Abstract

This study examined the gamma radiation shielding properties of test barium-lanthanum borate glasses having the composition (80 − x) B_2_O_3_–8TiO_2_–11ZnO–x BaO–1La_2_O_3_ (x = 26, 29, 32, and 35 mol%). The glasses are designated as Ba26La1, Ba29La1, Ba32La1, and Ba35La1, and were prepared from the melt-quenching method. The radiation shielding parameters, mass attenuation coefficient (MAC), linear attenuation coefficient (LAC), half-value layer (HVL), tenth-value layer (TVL), mean free path (MFP), and effective atomic number (Z_eff_), were evaluated by using Phy-X/PSD software, at energy (0.015–15 MeV). The results of this study indicate that an increased amount of BaO in the glasses improves the attenuation property of the glasses. For example, Ba35La1 had the highest LAC value of 186.392 cm^−1^ at 0.015 MeV. This suggests that the Ba35La1 glass has very good absorption for low energy gamma rays. Ba35La1 exceeds many lead-free shielding materials and achieves a comparable attenuation value to lead-based shielding glasses values with respect to attenuation coefficients for specific lead-based glasses, with energies relevant to diagnostic photons. Ba35La1 is a great lead-free candidate for medical and industrial radiation shielding applications. Some properties of the shielding characteristics in Ba35La1 strengthened this claim such as, the greatest shielding efficiency over the total energy spectrum was manifested by Ba35La1 < 0.1 MeV due to the photoelectric region, coupled with the K-edge discontinuity at 0.037 MeV as a result of the Ba absorption edge; thus confirming the role high-Z modifiers had in enhancing the attenuation coefficient results within the region of interest.

## Introduction

Radiation which is injurious to the health of those involved and to the environment should therefore be shielded to avoid such effects. Radiation can cause illness, possibly cancer, therefore finding good protective materials is of interest in such sectors as medical, energy, and space-related ones^1–4^.

Glass has been used as a radiation shielding media for years due to the fact that it is transparent and easy to shape. Moreover, where it was once utilized for fundamental shielding applications such as basic surface barriers, technological improvements have made it possible to create different types of glass having different compositions suitable for different shielding requirements^5–9^. Contemporary glasses are designed to protect from diverse sorts of the radiation, such as gamma ones and neutrons, to state several; at the same time, they are less heavy and have a more diverse range of designs. This evolution therefore increases the understanding of the glass as an all-round and efficient shield for different sorts of radiation in various industries^10–14^.

Improvements in the glass formulation in the recent past have boosted the radiation shielding ability of glass in a big way. They can vary the composition of the glasses and obtain the samples with the desired characteristics of shielding. The following developments make it possible to maximize density, mechanical properties, as well as the ability to attenuate radiation. The possibility of objective fine-tuning of glass compositions has extended the use of glass in very high radiance areas providing subsystems of better efficiency with minor deleterious effects to the environment. These innovations are creating the basis of the next generation, of shielding protection in healthcare, nuclear energy and aerospace industries among others^15–19^.

Boron is a foundational glass making material, while borate glass is the main source of composition in over 90% of commercial glasses. This is an extraordinary glass forming material as borate shows many unique properties. First, borate has a low melting temperature and excellent thermal stability, so it can even incorporate heavy (higher density) materials. When borate glass incorporates heavy metal oxides like lead oxide (PbO) or bismuth oxide (Bi_2_O_3_), density increases (including density)^20–22^. These examples of heavy metal oxides would fit into the gamma ray shielding applications. Alkali oxides that include lithium oxide (Li_2_O) or sodium oxide (Na_2_O) will also lower the melting temperature of glass^23–26^. Boron oxide glasses have received a considerable amount of research interest due to their heterogeneous compositions and interesting properties. Recently, borate glass has been explored in more suitable applications because of its unique properties, including broad optically transparent range, enhanced mechanical properties, and chemical stability. Zinc Oxide (ZnO) is directly related to the glass density and radiation attenuation. By enhancing radiation-shielding efficiency, ZnO has contributed to the development of protective materials intended for environments with high radiation levels^27,28^.

Boron Oxide (B_2_O_3_) is an intermediate for amorphous phase and a network former that when added to glass stabilizes it and improves its properties. B_2_O_3_ is used to enhance the physical properties in the glass and as such, it is essential in the production of superior shielding glass materials^29–31^. As a result, both in the incorporation of Calcium Oxide (CaO) and Magnesium Oxide (MgO) in the glass system, there is improved physical characteristics. Altogether, these oxides enhance the mechanical characteristics of the glass and enhance the aspects of radiation shielding for the glass required in critical protective applications^32,33^.

Experimental and simulation methods such as gamma-ray spectroscopy and Monte Carlo simulations are used to measure and assess the radiation shielding properties of glass. Methods such as these can assist in the determination of the best glass compositions in respect of the shielding specifications and demonstrate the efficacy of the glass that is produced following the experiments^34–37^. Increasing the number of glass compositions, in terms of lead, can also significantly benefit the environment and health risks. The use of lead is minimized while still providing adequate radiation shielding for patients and staff, which reduces the impact on the environment and on the health of people that the lead may still affect, meeting sustainability policies and guidelines.

Protective glass is highly useful, used in different industries, for instance, medicine, aviation, and in areas which involve the use of radiation. They are commonly employed in diagnostic shields, X-ray windows, and hot cells where transparency is coupled with resistance to radiation^38,39^. Compared to using concrete and lead sheets radiation shields made of glass have several advantages: transparent capability, lighter than the others and easier to form. As the comparison made here shows an optimization of glass compositions is capable of providing comparable or better radiation attenuation thereby offering prospective protective applications^40,41^. The aim of this study is to evaluate the influence of increasing BaO concentration on the shielding properties of new barium-lanthanum borate glasses based on borate (80 − x)B_2_O_3_–8TiO_2_–11ZnO–xBaO–1La_2_O_3_ (with x = 26, 29, 32 and 35 mol%), in orders to produce a lead-free glass that equals or exceeds the properties of the current lead-containing glasses over a broad range of gamma-ray energies.

## Materials and methods

### Glasses Preparation

**Fig. 1:**
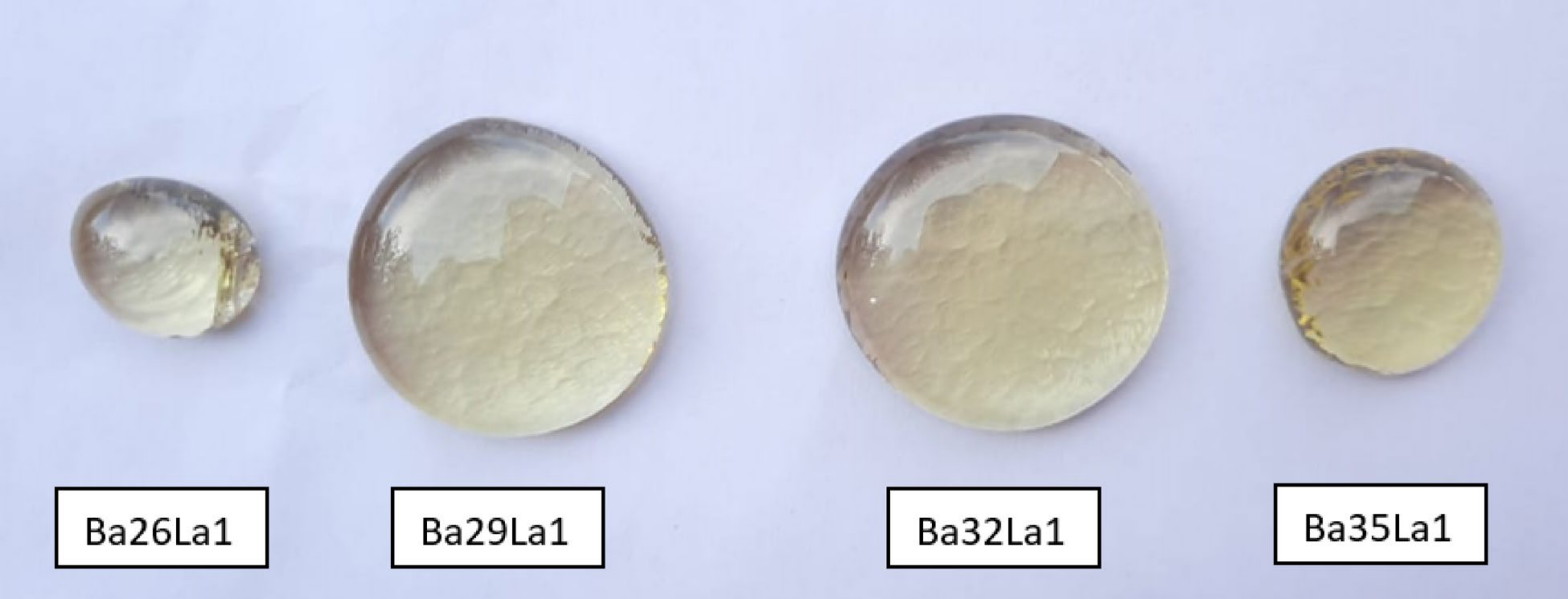
The photo of the B_2_O_3_-TiO_2_-ZnO-BaO-La_2_O_3_ glasses.

A new batch of glass samples with the chemical composition (80-x) B_2_O_3_-8TiO_2_-11ZnO-xBaO-1La_2_O_3_, x = 26, 29, 32 and 35 mol%) was prepared and characterized by Ba26La1, Ba29La1, Ba32La1, and Ba35La1, respectively. High purity oxides (> 99%) were used to prepare the glasses. The oxides used in this investigation were purchased from Loba Chemie PVT. LTD, India, while the La_2_O_3_ was purchased from Hebei Suoyi New Material Technology CO., LTD. (China). Each glass was prepared using the melt-quenching technique. The compositions are shown in Table 1 (in mol%). High-grade materials including B_2_O_3_, TiO_2_, BaO, ZnO and La_2_O_3_ were mixed together to obtain a homogeneous sample. The powders were melted in alumina crucibles at 1100 °C for 80–90 min until a homogeneous, bubble free melt was obtained. The molten glass was then cast onto a pre-heated stainless-steel plate before being annealed at 350 °C for 4 h to allow for the release of internal stresses to avoid crack formation. An image of glasses prepared is shown in Fig. 1. The density of the samples was calculated using Archimedes method.

**Table 1:** The chemical composition of the B_2_O_3_-TiO_2_-ZnO-BaO-La_2_O_3_ glasses.

Glass Code	B_2_O_3_	TiO_2_	ZnO	BaO	La_2_O_3_
Ba26La1	54	8	11	26	1
Ba29La1	51	8	11	29	1
Ba32La1	48	8	11	32	1
Ba35La1	45	8	11	35	1

### Radiation shielding performance

The software Phy-X/ PSD implements a computational analysis with properties of radiation shielding as shown in following reference^42,43^. The process is divided in three steps. First, by specifying the chemical and density of material, then define the energy area (broad range (0.015–15 MeV) or discrete values typically related to specific radiation sources). After editing, the possible options for analysis were selected with number of calculations for evaluating the properties of radiation shielding.

The mass attenuation coefficient (MAC, cm^2^/g) is a key property of a material that gives the likelihood of its effective attenuation (absorption/scattering) of photons (X-rays or gamma rays) per unit mass per unit area. It is defined as^44,45^:

1 $$\:MAC=\frac{LAC}{\rho\:}$$

The linear attenuation coefficient (LAC) is a physical parameter that can be used to quantify the ability of a medium to attenuate radiation as the radiation propagates through the medium. The LAC is calculated using; Eq. (2)^46^:

2 $$\:LAC\:=\:-ln(I/Io)\:/\:t,$$

where I is the intensity of gamma rays recorded with the constructed glass, Io is the initial intensity of gamma rays recorded without the constructed glass, and t is the thickness of the glass.

The Half-Value Layer (HVL) is defined as the thickness of the material necessary to reduce the intensity of a radiation beam (e.g., X-rays and gamma rays) to half (50%) of its original value and calculated according to Eq. (3)^47,48^:

3 $$\:HVL\:=\:0.693\:/\:LAC$$

The Mean Free Path (MFP, cm) is the average distance transcended by a photon before encountering an interaction (absorption, scattering, etc.), see Eq. (4).

4 $$\:MFP=\frac{1}{LAC\:}\:\:\:\:\:\:\:\:\:\:\:\:\:\:\:\:\:\:\:\:\:\:\:\:\:\:\:\:\:\:\:\:\:\:\:\:\:\:\:\:\:\:\:\:\:\:\:\:\:\:\:\:\:\:\:\:\:\:\:\:\:\:\:\:\:\:\:$$

The Tenth-Value Layer (TVL, cm) is the thickness of material that is needed to reduce the intensity of radiation levels to one-tenth (10%) of the original. It’s calculated by Eq (5).

5 $$\:TVL=\frac{{ln}\left(10\right)}{LAC}$$

## Results and discussion

The photon attenuation properties of the (x)B₂O₃ + 8TiO₂ + 11ZnO + (35 − x)BaO + 1La₂O₃ system of glasses were systematically analyzed while varying the content of B₂O₃ and BaO (x = 26, 29, 32–35 mol%). The structural design of this series of glasses utilizes oxides of low-Z and high-Z in order to optimize observable characteristics for radiation shielding while being processable. All calculations were made over a broad energy range (0.015–15 MeV) using Phy-X/PSD software^42^.

### The importance of glass composition to gamma radiation Attenuation

The composition of the glass, considering both the constituents and their quantities, has an important role in their characteristics regarding the attenuation of photons. Boron oxide (B₂O₃) (or boric oxide) was the main glass-former where these glasses would have good network flexibility and durability of the glass, but due to B’s low atomic number (Z = 5), would not be a key contributor to attenuation at excitation or therapeutic energy ranges. However, increasing the density of the glass (via a high-Z modifier as barium oxide (BaO) could improve attenuation characteristics significantly. In this regard, the substitution of B₂O₃ with BaO could be a fundamental exercise to amend the attenuation characteristics of the glass without losing stability in the glass-forming network^49–55^.

Barium was introduced incrementally into the batch, from 26 mol% to replacing with up to 35 mol%, and the cumulative increased proportion of BaO yielded increases in density and effective atomic number (Z_eff_). From these changes, we anticipate a major effect on the major attenuation measurements.

### Mass Attenuation coefficient (MAC)

**Fig. 2:**
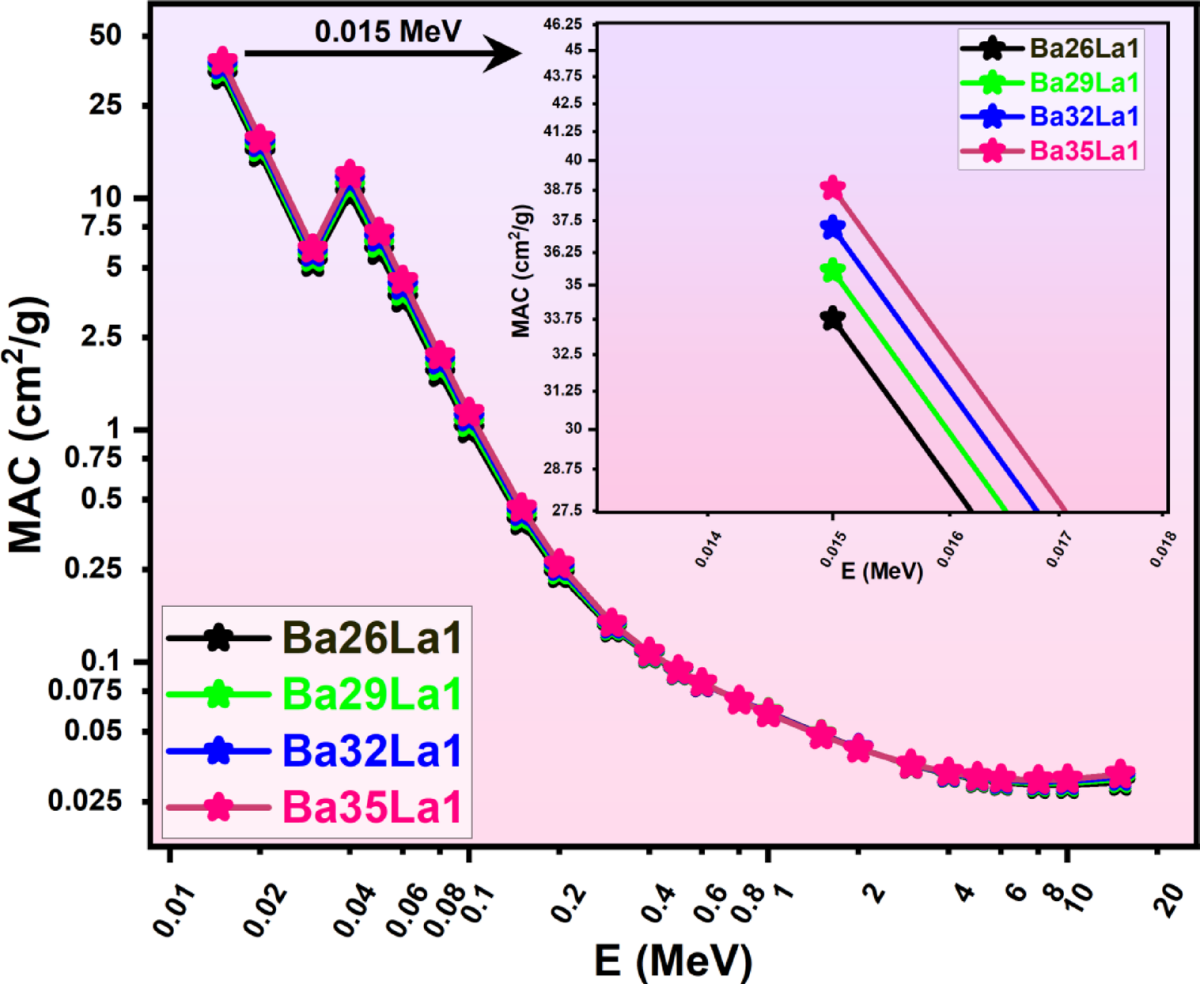
Mass attenuation coefficients (MAC, cm^2^/g) as a function of photon energy (E, MeV) for various barium-lanthanum (Ba-La) compounds (Ba26La1, Ba29La1, Ba32La1, Ba35La1) in the energy region from 0.015–15 MeV.

The calculated MAC values in Fig. 2. demonstrated the anticipated dependence on energy where at lower energies (< 0.1 MeV), MAC was influenced by the photoelectric effect producing steep attenuation curves; in the mid-energy range (0.1 -5 MeV), Compton scattering predominated; and at higher energies (> 5 MeV), pair production contributions emerged^56^.

One prominent characteristic from the MAC profile was the distinct discontinuity near 0.037 MeV that corresponded to the K-absorption edge of barium (Z = 56). The MAC discontinuity originated from the abrupt increase in the photoelectric cross-section as photon energy crossed the ionization energy for the Ba K-shell. The edge effect was more pronounced with increasing BaO concentration which suggested a dependence of the edge effect on the concentration of high-Z dopant^57,58^.

Among all samples, Ba35La1 exhibited higher MAC values over the entire energy range confirming that BaO contributed to increased photon interaction efficiency. This sample had approximately 20–25% higher MAC at diagnostic energy levels, compared to Ba26La1, illustrating the advantage of enrichment of barium in low-energy shielding decisions.

### Linear Attenuation coefficient (LAC), HVL, TVL, and MFP

**Fig. 3:**
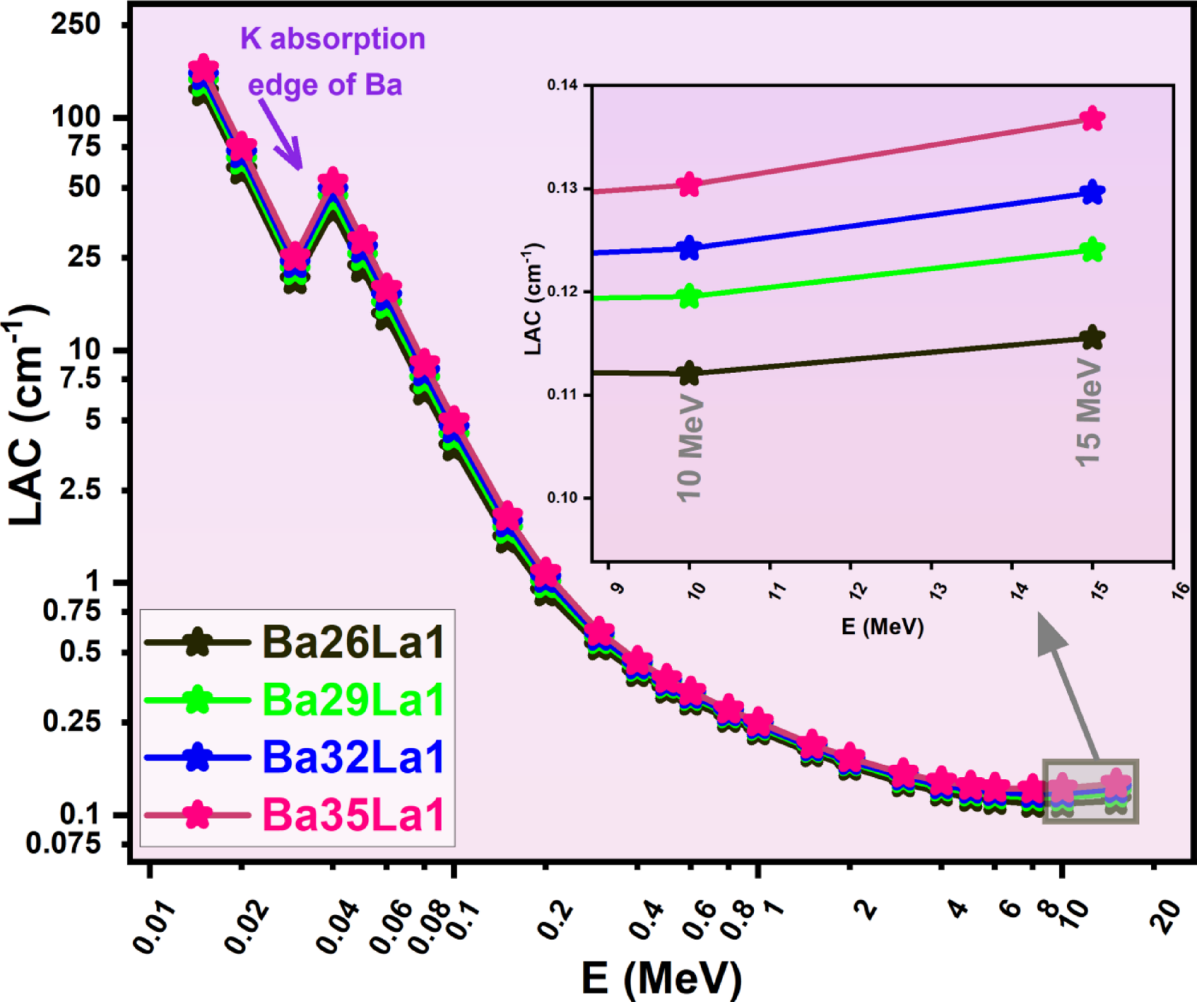
Linear attenuation coefficient (LAC, cm^−1^) as a function of photon energy (E, MeV) for various barium-lanthanum (Ba-La) compounds (Ba26La1, Ba29La1, Ba32La1, Ba35La1) in the energy region from 0.015–15 MeV.

The LAC was calculated by taking MAC and multiplying it with each sample’s density which was determined experimentally^59^. As anticipated, the LAC values in Fig. 3. adhered to a similar trend as MAC, but with better contrast as a result of the density differences. Again, the results confirmed that Ba35La1 had a greater shielding capability, as evidenced with the highest values of LAC.

**Fig. 4:**
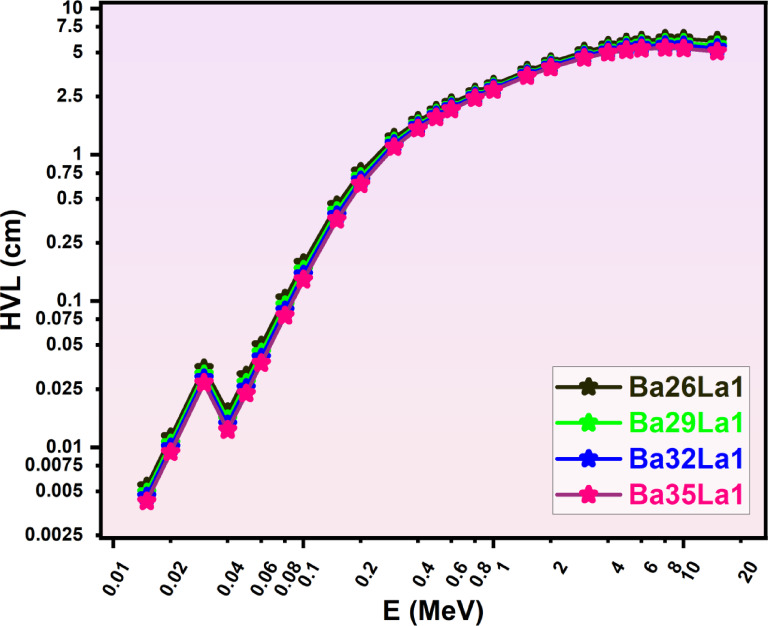
Half value-layer (HVL, cm) as a function of photon energy (E, MeV) for various barium-lanthanum (Ba-La) compounds (Ba26La1, Ba29La1, Ba32La1, Ba35La1) in the energy region from 0.015–15 MeV.

**Fig. 5:**
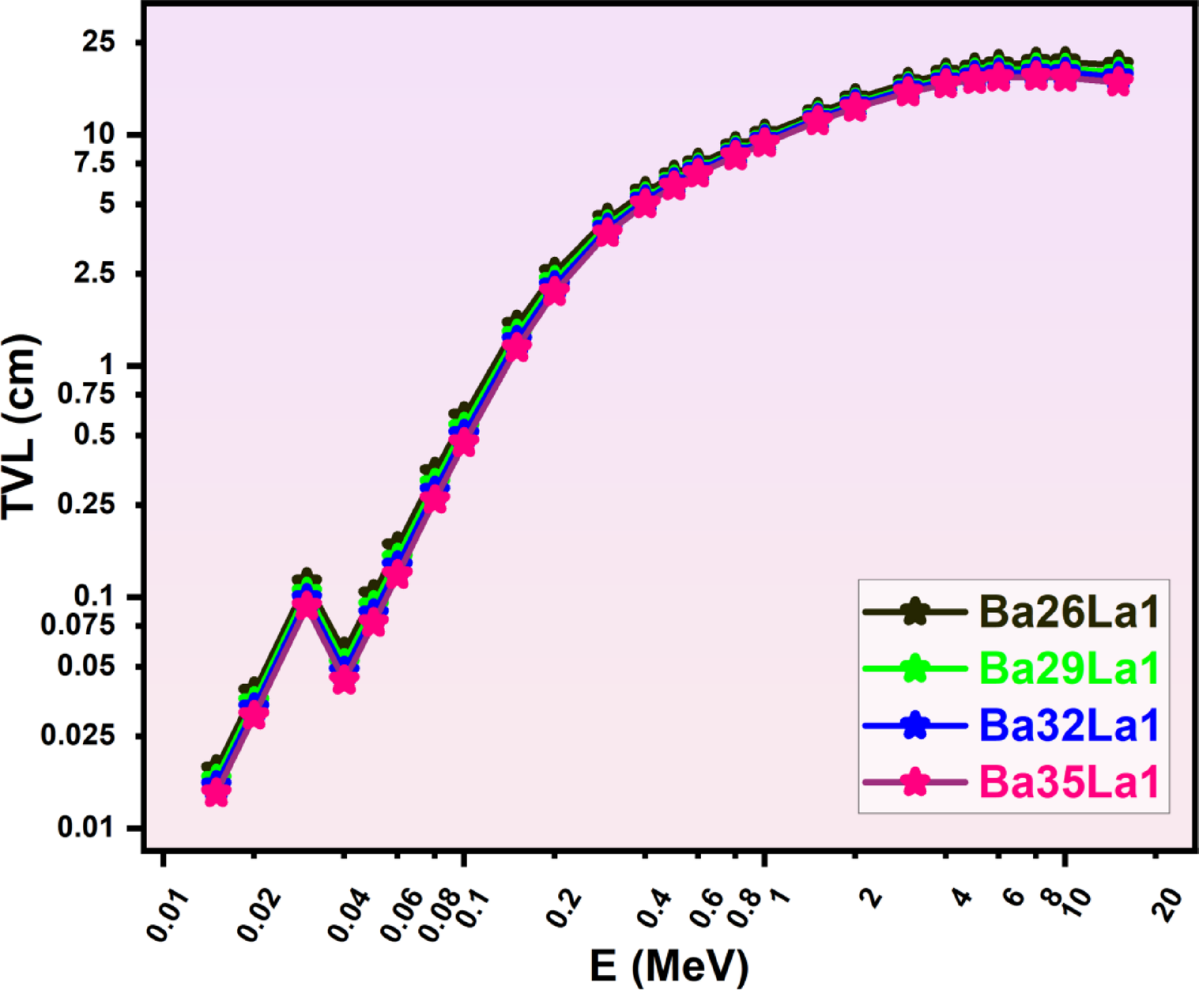
Tenth value layer (TVL, cm) as a function of photon energy (E, MeV) for various barium-lanthanum (Ba-La) compounds (Ba26La1, Ba29La1, Ba32La1, Ba35La1) in the energy region from 0.015–15 MeV.

**Fig. 6:**
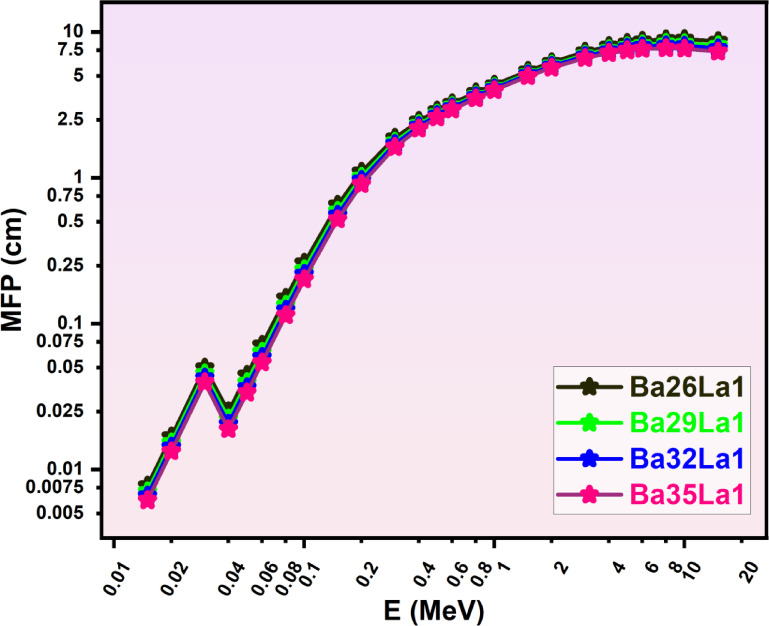
Mean free path (MFP, cm) as a function of photon energy (E, MeV) for various barium-lanthanum (Ba-La) compounds (Ba26La1, Ba29La1, Ba32La1, Ba35La1) in the energy region from 0.015–15 MeV.

After LAC was calculated, to evaluate shielding, it also had to be considered in terms of thickness, from a materials perspective. Consequently, the parameters of HVL (half-value layer, (Fig. 4)), TVL (tenth-value layer, (Fig. 5)), and MFP (mean free path, (Fig. 6)) were derived for consideration, these applicative sets represent configurations to consider shielding designs practically at different thickness levels^60^. Each of the parameters in Figs. 4, 5 and 6 presented were in a positive correlation with the photon energy of photons, which demonstrated that there is decreasing probability of interactions with increasing energies.

Barium glass with a composition of Ba35La1 had the lowest HVL (Fig. 4) and TVL (Fig. 5) for every energy investigated, which meant that Ba35La1 would attenuate the highest percentage of photons with the thinnest sections. Which is significant, for example the HVL for Ba35La1 at (0.6 MeV) was ~ 0.92 cm and was a ~ 1.25 cm HVL for Ba26La1 or 26% less thickness required.

### Effective atomic number (Z_eff_)

**Fig. 7:**
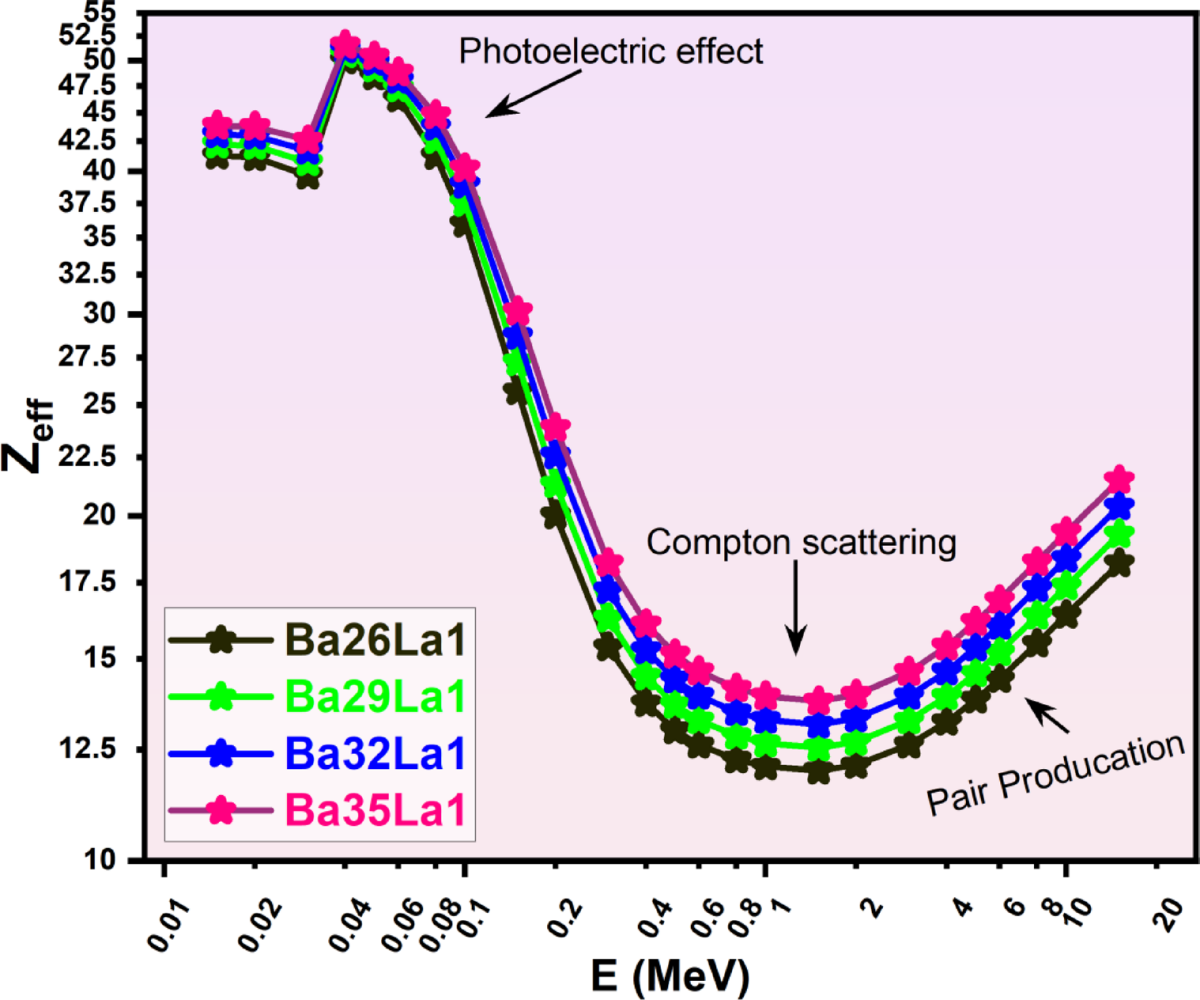
Effective atomic number (Z_eff_) as a function of photon energy (E, MeV) for various barium-lanthanum (Ba-La) compounds (Ba26La1, Ba29La1, Ba32La1, Ba35La1) in the energy region from 0.015–15 MeV.

The Z_eff_ profiles of the glasses were derived from MAC values by logarithmic interpolating the elemental cross-sections. The results are shown in Fig. 7. The Z_eff_, on the other hand, had a non-linear profile with respect to energy, which peaked in the low-energy region as a result of the predominance of photoelectric interactions. As energy increased, the Z_eff_ values plateaued in the Compton-dominated region.

The level of Z_eff_ increased with composition noticeably: Ba35La1 consistently exhibited the highest Z_eff_ values for all energy levels, measuring ~ 38 in the range of 0.05–0.1 MeV. Based on the current data, this is evidence that using BaO, instead of B₂O₃, improves the effective nuclear charge of the glass matrix and thus strengthens its attenuation.

### Comparative study to reported shielding materials

**Fig. 8:**
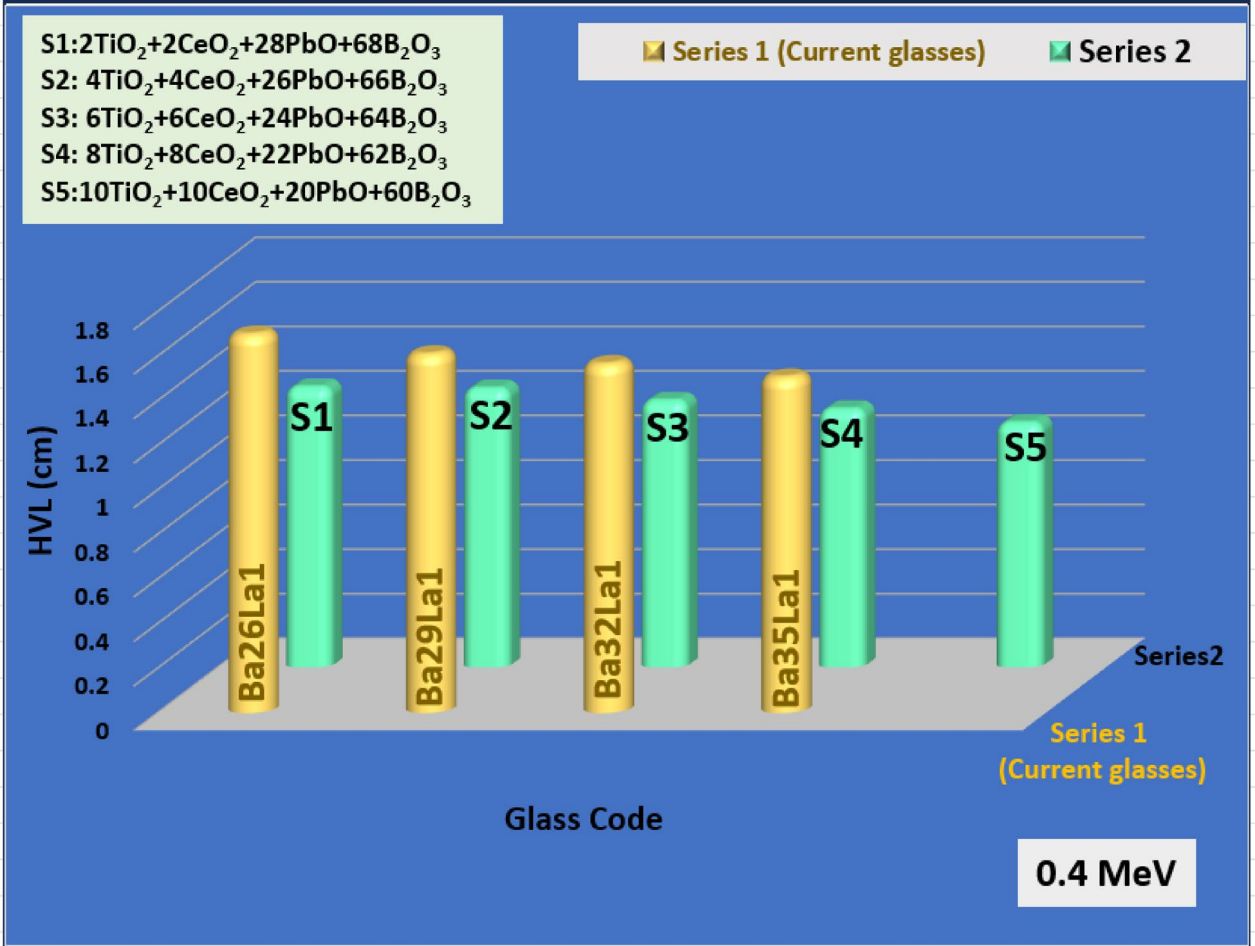
The comparison of the HVL of the BaLa glasses with the S glass series (TiO_2_-CeO_2_-PbO-B_2_O_3_) at gamma ray energy 0.4 MeV.

**Fig. 9:**
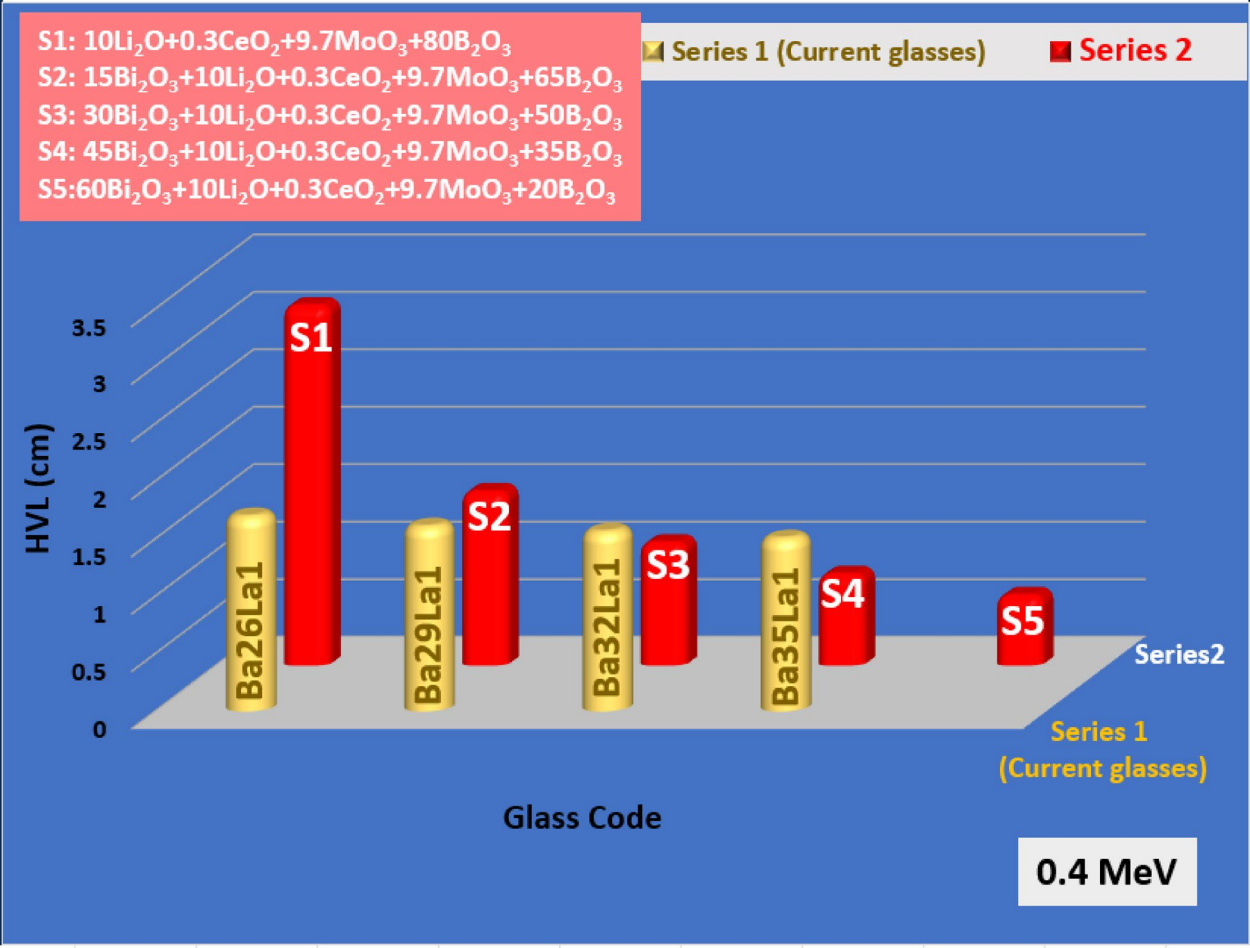
The comparison of the HVL of the BaLa glasses with the S glass series (Li_2_O-CeO_2_-MoO_3_-B_2_O_3_) at gamma ray energy 0.4 MeV.

**Fig. 10:**
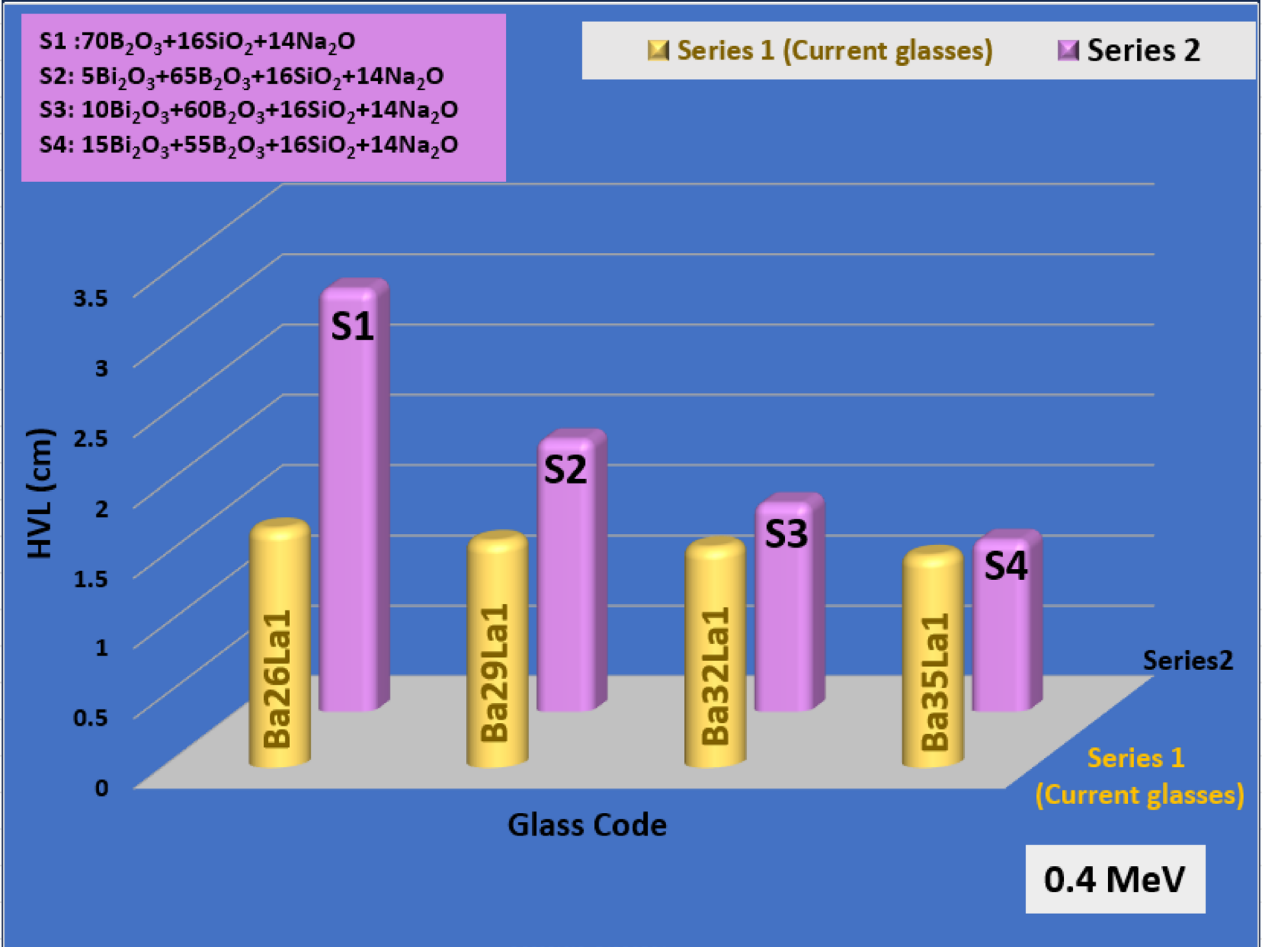
The comparison of the HVL of the BaLa glasses with the S glass series (B_2_O_3_-SiO_2_-Na_2_O) at gamma ray energy 0.4 MeV.

To assess the performance of the new glass system, the attenuation values of Ba35La1 were compared to the literature values of lead-free systems such as Bi₂O₃–Li₂O–B₂O₃ (Fig. 8), CeO₂–PbO–SiO₂ (Fig. 9), and TeO₂–ZnO–Na₂O types of glasses (Fig. 10)^61–63^. Ba35La1 had equivalent or better MAC and lower HVL values reported at 0.4 MeV, and further benefited from lower toxicity, thermal stability, and tunable glass structure.

Most importantly, Ba35La1 has equivalent performance to lead-based glasses in the medium-energy regime but does not include the handling and environmental issues seen with Pb products, making it a high-potential glass for sustainable radiation shielding material for medical and nuclear applications, particularly in cases where transparency, reduced weight, and/or recycling are preferred.

The findings exhibit that verifying, the systematic addition of BaO to the glassy matrix of borate–titanate–zincate glass enhances the gamma-ray shielding effectiveness, especially in the diagnostic and therapeutic energy windows. The relations noted in the attenuation parameters are well grounded in theoretical mechanisms of photon interaction and fit appropriately into our expectations of high-Z dopant performance. This forms a basis for the rational design of next-generation, lead-free, optically clear shielding materials for a multitude of technology opportunities.

## Conclusion

The present study shows that increasing the BaO content in barium-lanthanum borate glasses improved the properties of gamma radiation shielding, where Ba35La1 (35 mol% BaO) exhibited the greatest attenuation over a wide energy range (0.015–15 MeV). The increased shielding performance can be attributed to the photoelectric effect predominating at low energies, Compton scattering occurring at intermediate energies, and pair production becoming an important attenuation effect at high energies. There is an obvious break point at 0.037 MeV, which is due to the K-edge effect because of barium’s high atomic number. The Ba35La1 composition performed better overall than lead-free compositions while avoiding the environmental and toxicity problems of lead-based alternatives. In conclusion, the Ba35La1 borate glass demonstrates great potential as a sustainable and high-performance shielding material for medical, industrial, and nuclear applications, while also having the advantages of being a lightweight, potentially transparent, and recyclable glass material. While this paper investigates the theoretical radiation shielding properties of the prepared glasses over a wide energy range, it is recommended to extend this work by evaluating the optical properties, structural properties, as well as radiation shielding using experimental methods.

## Data Availability

The data presented in this study are available on request from the corresponding author.
